# HMGB1 downregulates DDX3 to activate the MAPK pathway, promoting the progression of colorectal cancer

**DOI:** 10.1038/s41417-025-00963-z

**Published:** 2025-09-20

**Authors:** Lin Ma, Meng Xu, Shaoxian Xu, Xueyan Guo, Wei Zong, Xi Zhao, Zi Yang, Guisheng Liu, Lin Shen

**Affiliations:** 1 Department of Gastroenterology, Shaanxi Provincial People’s Hospital, Xi’an, China; 2 Department of General Surgery, The Second Affiliated Hospital of Xi’an Jiaotong University, Xi’an, China; 3 Department of Dermatology, The Second Affiliated Hospital of Xi’an Jiaotong University, Xi’an, China; 4 Department of Hepatobiliary Surgery, Shaanxi Provincial People’s Hospital, Xi’an, China

**Keywords:** Cancer genetics, Gene expression

## Abstract

High mobility group box 1 (HMGB1) has been implicated in the development of various cancers, but its role in colorectal cancer (CRC) remains poorly understood. This study investigated the role of HMGB1 in CRC progression, particularly through its interaction with DEAD-box helicase 3 (DDX3), which, as demonstrated by our previous research, regulates CRC via the MAPK pathway. We analysed HMGB1 expression in CRC using public databases and tissue microarrays and detected significantly higher expression in CRC tissues than in normal tissues, which was associated with poor prognosis. HMGB1 expression was knocked down in the SW480 and HCT116 cell lines using siRNA and lentiviral vectors, and this knockdown inhibited CRC cell proliferation, migration, invasion, and adhesion, as confirmed by both in vitro and in vivo experiments. Molecular analyses revealed reduced phosphorylation of Erk1/2, c-Jun, and Elk1, along with decreased β-catenin and Snail expression and increased E-cadherin expression. Coimmunoprecipitation assay results further confirmed the interaction between HMGB1 and DDX3. These findings suggest that HMGB1 is an oncogene in CRC that promotes tumour progression through the MAPK pathway by downregulating DDX3. These findings highlight HMGB1 as a potential therapeutic target in CRC.

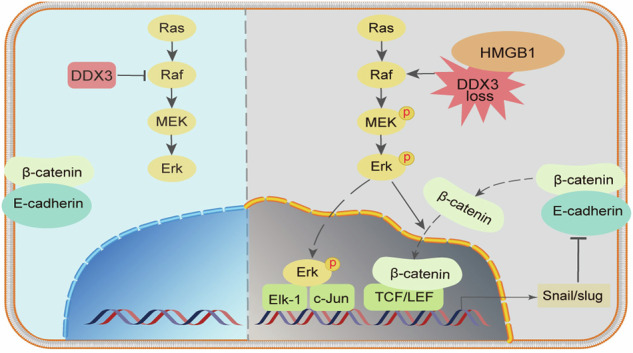

## Introduction

Colorectal cancer (CRC) is a malignancy that affects the colorectal mucosal epithelium and arises due to the interplay of genetic and environmental factors [1, 2]. Data from Global Cancer Statistics 2020 revealed that CRC was the third most commonly diagnosed cancer and the second leading cause of cancer-related death worldwide [3]. Early-stage CRC may be asymptomatic, whereas advanced-stage CRC is associated with a poorer prognosis because of the greater likelihood of recurrence or metastasis [4]. Additionally, the economic burden of CRC worldwide is significant [5]. Consequently, challenges related to the early detection and management of CRC, particularly in patients with advanced disease, remain.

The high mobility group box (HMGB) protein family, consisting of four members—HMGB1, HMGB2, HMGB3, and HMGB4—plays critical roles in regulating nucleosome dynamics [6, 7], DNA damage repair, telomere stability [8], and other essential genetic functions [9]. Recent studies have shown that HMGB1 is overexpressed in a variety of cancers, including CRC. Elevated HMGB1 expression is associated with lymphatic metastasis, distant metastasis, and poor prognosis in CRC patients [10–13]. However, the precise underlying mechanism remains incompletely understood.

In this study, we evaluated HMGB1 expression in CRC through an analysis of a public tumor database and two paired tissue microarrays (TMAs). We investigated the correlation between HMGB1 expression levels and various clinicopathological factors, as well as the effect of HMGB1 expression on patient survival. Through cellular experiments, we confirmed the role of HMGB1 in promoting the malignant behavior of tumor cells. Building on our previous research, which demonstrated that DEAD-box helicase 3 (DDX3) suppresses CRC progression via the MAPK pathway [14], we further explored the relationship between HMGB1 and DDX3. Our findings, confirmed by coimmunoprecipitation (co-IP) assay results, show that knockdown of HMGB1 results in the upregulation of DDX3 expression. This, in turn, inhibits CRC progression through the classical MAPK pathway and suppresses epithelial‒mesenchymal transition (EMT).

## Materials and methods

### Cell lines and cell culture

The human colon cancer cell lines HCT116 and SW480 were purchased from the Cell Bank of the Chinese Academy of Sciences (Shanghai, China). All cell lines were recently authenticated using short tandem repeat profiling and confirmed to be free of mycoplasma contamination. Cells were cultured in Dulbecco’s Modified Eagle Medium (DMEM; Gibco, Grand Island, NY, USA) supplemented with 10% fetal bovine serum (FBS; Gemini Bio-products, West Sacramento, CA, USA), and maintained at 37 °C in a humidified 5% CO₂ atmosphere.

### Immunohistochemistry (IHC) and immunostaining intensity assessment

For IHC, TMAs comprising 101 CRC tissues and 79 adjacent nontumour tissues were purchased from Outdo Biotech Co., Ltd, Shanghai, China (HColA180Su15). IHC staining was performed using an IHC SP Kit (#SP-9001; ZSGBBIO, Guangzhou, China) according to the manufacturer’s instructions. The primary antibodies that were used were anti-HMGB1 (1:50; 10829-1-AP; Proteintech, Chicago, IL, USA), anti-E-cadherin (1:400; 3195; CST, Darmstadt, Germany) and anti-p-Erk1/2 (1:400; 4370; CST). TMA staining was independently evaluated by two pathologists. The immune activity was categorized into five grades (percentage score) on the basis of the percentage of stained cells: negative staining (0), 1–25% (1), 26–50% (2), 51–75% (3), and >75% (4). Additionally, staining intensity was classified into four grades (intensity score): negative (0), weak (1), medium (2), and strong (3). The final overall histological score was calculated by multiplying the percentage score by the intensity score.

### siRNA transfection

Two small interfering RNAs (siRNAs), labeled si-1 and si-2, that target HMGB1, along with a negative control siRNA (si-NC), were purchased from GenePharma Co., Ltd (Shanghai, China). Cellular transfection of the siRNAs was performed using Lipofectamine 2000 (11668030; Invitrogen, Waltham, MA, USA) according to the manufacturer’s instructions. Then, the cells were harvested 48 h post transfection. The efficacy of the transfection was then evaluated by Western blotting analysis. Si-2 (with greater knockdown efficiency) was selected for functional assays in the HCT116 and SW480 lines to ensure phenotype reproducibility across distinct genetic backgrounds.

### CCK8 and colony formation assays

After siRNA transfection, the cells were resuspended in complete growth medium and plated in 96-well culture plates at a density of 15,000 cells/mL (3000 cells per well in 200 μL of medium). The plates were then incubated overnight at 37 °C under standard culture conditions (5% CO₂ and 95% humidity). At 24-h intervals, one plate was collected for analysis. In accordance with the manufacturer’s protocol, 10 µl of CCK-8 reagent (Beyotime, Shanghai, China) diluted in 90 µl of DMEM was added to each well. After a one-hour incubation, the absorbance was measured at 450 nm to assess cell viability. For the colony formation assay, cells were seeded in 6-well plates at a density of 1 × 10³ cells per well and maintained in culture for 14 days. After the incubation period, cells were fixed with 4% paraformaldehyde and stained with 5% (w/v) crystal violet solution. Colony quantification was performed by counting visually distinct colonies under standardized conditions. A countable colony was rigorously defined as a cluster containing ≥50 viable cells after the 14-day culture period, satisfying both morphological criteria (exhibiting contiguous growth) and minimum size requirement (>0.5 mm in diameter).

### Transwell migration and invasion assays

Cell migration and invasion assays were performed using Transwell plates (Kennebunk, ME, USA). For the migration assay, 1 × 10^4^ cells were suspended in medium supplemented with 10% FBS and seeded into the upper chambers of Transwell plates. Subsequently, 600 µl of DMEM supplemented with 30% FBS was added to the lower chamber. After 24 h of incubation at 37 °C in a 5% CO₂ atmosphere, the cells that had migrated to the lower chamber were fixed with 4% paraformaldehyde and stained with 1% crystal violet for 20 min. In the invasion assay, the upper chamber membrane was precoated with Matrigel (BD Biosciences, Franklin Lake, NJ, USA). Following a 48-h incubation period, cells that had migrated to the lower surface of the membrane were fixed with 4% paraformaldehyde for 15 min at room temperature, then stained with 5% (w/v) crystal violet solution for 20 min. After washing with phosphate-buffered saline (PBS), the stained cells were imaged using an inverted light microscope (Nikon Eclipse Ti, 10× objective). For quantitative analysis, five randomly selected fields (200× magnification) from each membrane were captured and analyzed using ImageJ software (version 1.53) with consistent threshold settings across all samples to ensure unbiased quantification and experimental reproducibility.

### Cell adhesion assay

Prior to cell plating, each well of each 96-well plate was coated with 30 µL/well of Matrigel matrix (#354234 BD Biosciences Franklin Lake, NJ, USA) that had been thawed on ice and diluted 1:1 in cold DMEM. The coated plates were incubated at 37 °C for 2 h to allow matrix polymerization. Subsequently, the cells were seeded into the 96-well plates and cultured for 40 min, followed by addition of 100 µl of medium containing ~1 × 10^4^ cells to each well. After 1 h of incubation, the cells were fixed, stained, and imaged using a microscope.

### Western blotting

Total protein was extracted from the cells with RIPA buffer (P0013C; Beyotime, Shanghai, China) supplemented with protease inhibitors (B14001; Bimake, Houston, TX, USA). Following protein extraction and quantification using the BCA method, equal amounts of total protein (20 μg per lane) were separated by SDS-PAGE (P0012A; Beyotime, Shanghai, China) using a 5% stacking gel (pH 6.8) and 6–12% separating gel (pH 8.8), with specific concentrations optimized for target protein molecular weights (6% for >100 kDa, 10% for 30–100 kDa, 12% for <30 kDa). Electrophoresis was performed at 80 V through the stacking gel and 120 V through the resolving gel. Proteins were then electrophoretically transferred to polyvinylidene difluoride (PVDF) membranes (1060023; GE Amersham, Chicago, IL, USA) at 100 V for 90 min at 4 °C. The membranes were blocked with 5% nonfat milk in Tris-buffered saline with Tween® 20 (TBST) to prevent nonspecific binding and then incubated overnight at 4 °C with the following primary antibodies: anti-HMGB1 (1:500; 10829-1-AP; Proteintech, Chicago, IL, USA), anti-DDX3 (1:1000; 11115-1-AP; Proteintech), anti-Erk1/2 (1:1000; 4659; CST, Darmstadt, Germany), anti-p-Erk1/2 (1:2000; 4370; CST), anti-c-Jun (1:1000; AF6090; Affinity Biosciences, Cincinnati, OH, USA), anti-p-c-Jun (Ser73) (1:1000; AF3095; Affinity Biosciences), anti-Elk1 (1:1000; AF6212; Affinity Biosciences), anti-p-Elk1 (Ser383) (1:1000; AF3212; Affinity Biosciences), anti-E-cadherin (1:1000; 3195; CST), anti-p-β-catenin (Ser675) (1:1000; 4176; CST), anti-Snail (1:1000; ab216347; Abcam, Cambridge, UK), and anti-β-actin (1:1000; ab8227; Abcam). After being washed with TBST, the membranes were incubated with goat anti-rabbit (1:6000; EK020; Zhuangzhi, Xi’an, China) and goat anti-mouse (1:6000; EK010; Zhuangzhi) antibodies for 1 h at room temperature. Following further washes, specific protein bands were detected with an ECL (P0018AFT, Beyotime, Shanghai, China) substrate. Band intensities were quantified using ImageJ (version 1.53) and normalized to the loading control.

### Lentiviral infection

HMGB1 knockdown was achieved using lentiviral delivery of a specific shRNA (GV493 vector, contract #GISL0350526; sequence provided in Supplementary Table 1) from GeneChem (Shanghai). HMGB1 overexpression used GV358 vector (contract #GOSL0106841, GeneChem, Shanghai, China). Stable cell lines were established through 24-h infection (8 μg/mL polybrene) followed by 4-week puromycin selection (2 μg/mL), with efficiency verified by qPCR and Western blotting.

### Co-IP assay

Total protein was extracted from the cells via NP-40 lysis buffer (ST2048; Beyotime, Shanghai, China). The protein concentration was determined using the BCA method. Subsequently, anti-HMGB1 (1:500, 10829-1-AP; Proteintech, Chicago, IL, USA) and anti-DDX3 (1:1000; 11115-1-AP; Proteintech) antibodies were separately added to the 1000 µg total protein mixture, and the mixture was incubated overnight at 4 °C with gentle shaking. On the following day, 30 μL of protein A/G beads (P2180S; Beyotime, Shanghai, China) were added, and the mixture was shaken for 2 h at 4 °C. The magnetic beads were then collected, suspended in NP-40 lysis buffer and loading buffer, and denatured at 95 °C for 5 min. The sample mixture was subsequently collected using a magnetic separator. Protein interactions were analysed by Western blotting.

### Tumorigenesis experiment in BALB/c nude mice

The animal experiment was approved by the Animal Ethics Committee of Shaanxi Provincial People’s Hospital. Male BALB/c nude mice (6 weeks old) were obtained from the Experimental Animal Center of Xi’an Jiaotong University (Xi’an, China). Power analysis was performed a priori using G*Power 3.1 (α = 0.05, β = 0.20), which indicated that a sample size of *n* = 4 per group would provide >80% statistical power to detect significant differences. In accordance with statistical power calculations and ethical requirements, four nude mice were randomly allocated to each experimental group using a random number table method in this non-blinded study. For each mouse, 1 × 10⁶ CRC cells were subcutaneously injected or administered via tail vein injection. The mice were housed at the Specific Pathogen-Free Animal Center of Xi’an Jiaotong University. The subcutaneous tumor diameter was measured weekly, and the animals were euthanized after 6 weeks. Tumors were then isolated, weighed, and fixed in paraffin. The tumor sections were subjected to haematoxylin and eosin (H&E) and IHC staining. The tumor volume was calculated using the following formula: tumor volume (mm^3^) = [length (mm) × width (mm)^2^] π/6.

### Statistical analysis

All the statistical analyses were performed using GraphPad Prism 5 and SPSS 22.0 software. One-way analysis of variance (ANOVA), the chi-square test, or Fisher’s exact test was used to assess the relationships between HMGB1 expression and clinicopathological parameters. Additionally, *t* tests and ANOVA were used to analyse continuous data. Kaplan‒Meier analysis and the log-rank test were performed to evaluate the correlation between HMGB1 expression and overall survival and recurrence-free survival of individuals with CRC. Multivariate survival analysis was conducted using the Cox proportional hazards model. All the statistical tests were two-tailed. The experimental procedures were conducted in a blinded and randomized manner. The data are presented as the means ± standard deviations (SDs) of three independent experiments. Statistical significance was defined as *p* < 0.05.

## Results

### The expression of HMGB1 is increased in CRC tissues and correlates with clinicopathological parameters and patient survival

**Fig. 1:**
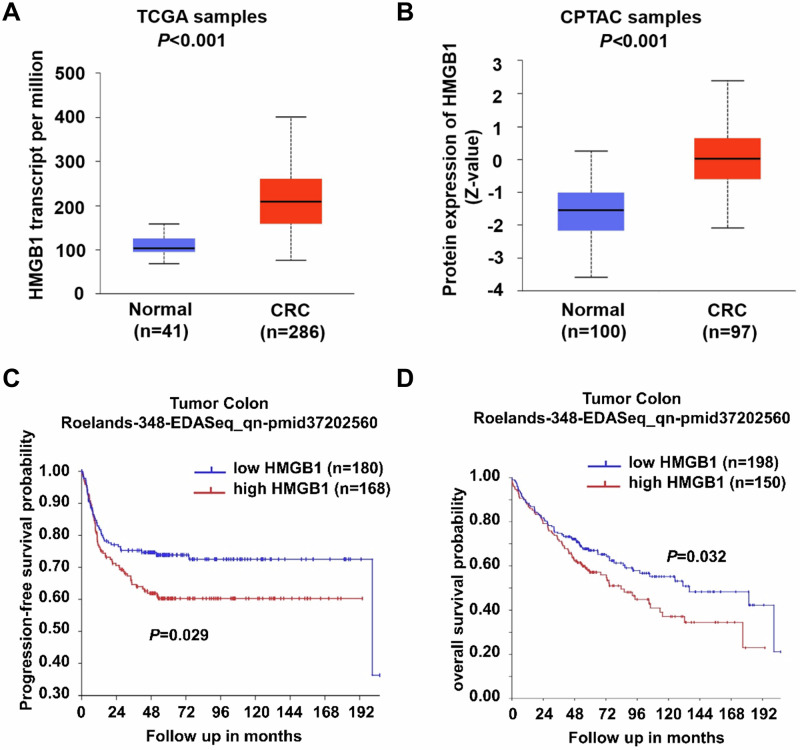
Bioinformatics analysis of HMGB1 expression and its significance in CRC. **A** Expression of HMGB1 mRNA in normal and CRC tissues by TCGA database (*p* < 0.001); **B** Expression of HMGB1 protein in normal and CRC tissues by the CPTAC database (*p* < 0.001); **C** Correlation between HMGB1 mRNA expression and progression-free survival by the R2 database. **D** Correlation between HMGB1 mRNA expression and overall survival by the R2 database.

To investigate the expression of HMGB1 across different tissues as well as its prognostic significance, we analysed data from the University of Alabama at Birmingham Cancer Data Analysis Portal (UALCAN). Our analysis revealed that HMGB1 expression was significantly elevated in CRC tissues compared with normal tissues at both the mRNA (Fig. 1A) and protein levels (Fig. 1B). Additionally, utilizing data from the R2: Genomics Analysis and Visualization Platform, we observed that patients with high HMGB1 expression had shorter relapse-free survival (Fig. 1C) and overall survival (Fig. 1D) times than those with low HMGB1 expression.

**Fig. 2:**
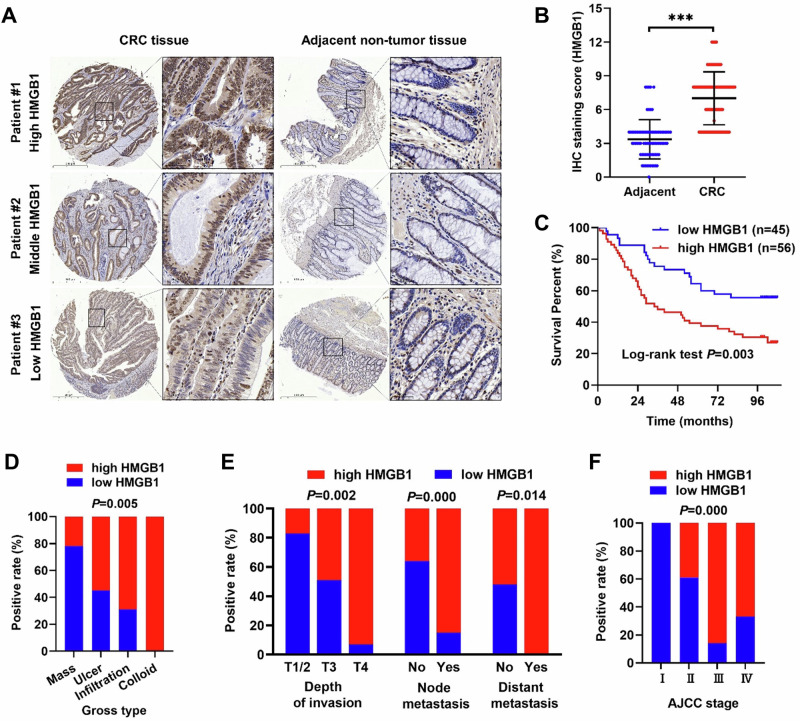
Relationship between HMGB1 expression and clinicopathological parameters in CRC. **A** Representative images of IHC staining of HMGB1 in CRC TMA; **B** HMGB1 expression in tumor tissues and adjacent normal tissues. (the expression level of HMGB1 was evaluated semi quantitatively by staining intensity). **C** Kaplan–Meier survival analysis showed that the high expression level of HMGB1 was associated with poor overall survival in CRC patients (*n* = 101; low expression score: 0–6, high expression score: 8–12, *p* = 0.003); **D** The relationship between HMGB1 expression and gross type (*p* = 0.005); **E** The relationship between HMGB1 expression and tumor depth of invasion (*p* = 0.002), lymph node metastasis (*p* < 0.001), distance metastasis (*p* = 0.014); **F** The relationship between HMGB1 expression and AJCC stage (*p* < 0.001).

Subsequently, IHC staining was performed on a TMA containing samples from 101 CRC patients and 79 adjacent normal tissues. The staining revealed nuclear expression of HMGB1 (Fig. 2A), which was significantly upregulated in CRC tissues compared with adjacent tissues (Fig. 2B and Table 1). On the basis of clinical pathologist scoring, samples were categorized into high and low HMGB1 expression groups, and statistical analysis was performed in conjunction with various clinicopathological parameters. Our findings indicated that high HMGB1 expression was associated with changes in tumor gross type (Fig. 2D), depth of invasion, lymph node metastasis, distant metastasis (Fig. 2E), and the American Joint Committee on Cancer (AJCC) stage (Fig. 2F and Table 2). Kaplan–Meier survival curves revealed that high HMGB1 protein levels in CRC patients were associated with significantly shorter overall survival times than low HMGB1 protein levels (Fig. 2C and Table 3).

**Table 1:** Comparison of HMGB1 expression between tumor tissues and adjacent non-tumor tissues.

Group	*N*	HMGB1 expression		*P* value
		Low	High	
Adjacent non-tumor tissues	79	74 (93.67%)	5 (6.33%)	<0.001
CRC tissues	101	45 (44.55%)	56 (55.45%)	

*CRC* colorectal cancer, *N* number of cases.

**Table 2:** Association of HMGB1 expression with clinicopathological parameters of patients with CRC.

Parameter	*N*	HMGB1 expression		*P* value
		Low	High	
Gender				0.173
Female	44	16	28	
Male	56	28	28	
Age				0.800
≤65	38	17	21	
>65	57	24	33	
Tumor location				0.569
Left-side	43	20	23	
Right-side	54	22	32	
Gross type				0.005**
Mass	18	14	4	
Ulcer	44	20	24	
Infiltration	35	11	24	
Colloid	3	0	3	
Tumor size (cm)				0.472
≤5	49	20	29	
>5	50	24	26	
Histologic type				0.673
Tubular	85	39	46	
Mucinous	15	6	9	
Pathology grade				0.706
I-II	54	25	29	
III	47	20	27	
Depth of invasion				0.002**
T1/2	6	5	1	
T3	77	39	38	
T4	14	1	13	
Node metastasis				0.000***
No	61	39	22	
Yes	39	6	33	
Distant metastasis				0.014*
No	94	45	49	
Yes	7	0	7	
AJCC stage				0.000***
I	5	5	0	
II	56	34	22	
III	36	5	31	
Ⅳ	3	1	2	

*AJCC* the American Joint Committee on Cancer, *N* number of cases. *p < 0.05; **p < 0.01; ***p < 0.001.

**Table 3:** Kaplan–Meier survival analysis with log-rank test.

HMGB1 expression	*N*	Mortality	Survival time (month)		*P* value
			average	95% CI	
Low	45	20 (44.4%)	76.9	65.9–87.9	0.003
High	56	40 (71.4%)	53.1	42.5–63.7	

*N* number of cases.

Furthermore, we performed univariate and multivariate Cox regression analyses to determine whether HMGB1 expression is an independent prognostic factor in CRC. Univariate regression analysis revealed that factors such as age, tumor location, histologic type, depth of invasion, lymph node metastasis, distant metastasis, AJCC stage, and HMGB1 expression significantly influenced survival (*p* < 0.05) (Table 4). Multivariate regression analysis further confirmed that age, tumor location, and HMGB1 expression were independently associated with survival (*p* < 0.05) (Table 5). These findings suggest that HMGB1 expression serves as a negative prognostic factor in CRC, independent of other clinical parameters.

**Table 4:** Univariate analysis of association between clinicopathological parameters and survival of CRC patients.

Parameter	*N*	HR	95% CI for HR	*P* value
Gender				0.888
Female	44			
Male	56	1.038	0.619–1.740	
Age				0.004**
≤65	38			
> 65	57	2.325	1.304–4.145	
Tumor location				0.023*
Left-side	43			
Right-side	54	1.882	1.091–3.246	
Gross type				0.125
Mass	18			
Ulcer	44	1.199	0.557–2.580	0.643
Infiltration	35	1.598	0.739–3.454	0.234
Colloid	3	4.371	1.163–16.433	0.029
Tumor size (cm)				0.781
≤5	49			
> 5	50	0.930	0.558–1.551	
Histologic type				0.025*
Tubular	85			
Mucinous	15	2.067	1.094–3.903	
Pathology grade				0.117
I-II	54			
III	47	1.501	0.903–2.492	
Depth of invasion				0.018*
T1/2	6			
T3	77	2.128	0.515–8.785	0.297
T4	14	4.875	1.088–21.855	0.038
Node metastasis				0.000***
No	61			
Yes	39	2.611	1.557–4.376	
Distant metastasis				0.000***
No	94			
Yes	7	8.233	3.526–19.226	
AJCC stage				0.000***
I	5			
II	56	3.222	0.438–23.697	0.250
III	36	7.255	0.983–53.543	0.052
Ⅳ	3	70.203	6.729–732.401	0.000
HMGB1 expression				0.004**
Low	45			
High	56	2.225	1.298–3.813	

*AJCC* the American Joint Committee on Cancer, *CI* confidence interval, *HR* hazard ratio, *N* number of cases. *p < 0.05; **p < 0.01; ***p < 0.001.

**Table 5:** Multivariate analysis of correlation between clinicopathological parameters and survival of CRC patients.

Parameter	Freedom	B	Wald	HR	95% CI for HR	*P* value
Age	1	0.840	7.261	2.315	1.257-4.264	0.007**
Tumor location	1	0.626	4.933	1.870	1.076-3.250	0.026*
HMGB1 expression	1	0.782	7.107	2.185	1.230-3.881	0.008**

*B* regression coefficient, *CI* confidence interval, *HR* hazard ratio. HR = Exp (B). *p < 0.05; **p < 0.01.

### The downregulation of HMGB1 inhibits the proliferation, migration, and invasion of CRC cells in vitro

**Fig. 3:**
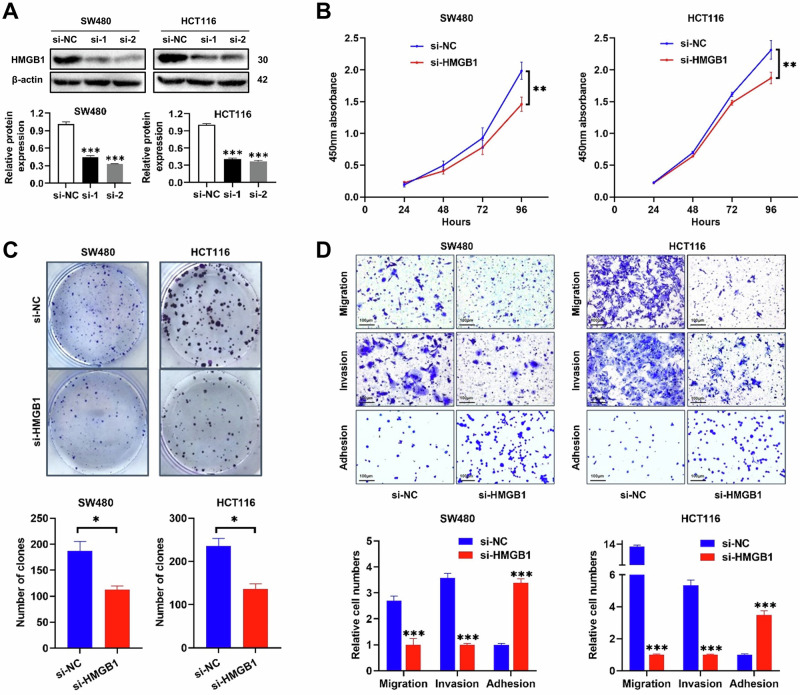
The decreased expression of HMGB1 inhibited cell proliferation, migration and invasion in CRC cell lines. **A** Decreased expression level of HMGB1 in si-1 and si-2 compared with si-NC in SW480 cells (left panel, ***p < 0.001) and HCT116 cells (left panel, ***p < 0.001); **B** Decreased absorbance value at 450 nm in si-HMGB1 cells compared with si-NC in CCK8 assay in SW480 and HCT116 cells (**p < 0.01); **C** Representative images demonstrating the inhibition of colony formation by decreased HMGB1 expression, along with a bar graph depicting the analysis of colony numbers (*p < 0.05); **D** The relative numbers of migrating, invading, and adhering SW480 and HCT116 cells were measured by Transwell assay and adhesion assay (***p < 0.001). All graphs are drawn from the mean ± SD of three independent replicates.

To investigate the role of HMGB1 in CRC cell function, we selected the SW480 and HCT116 cell lines as experimental models. We utilized two distinct siRNAs, namely, si-1 and si-2, to downregulate HMGB1 expression in CRC cells, and we validated the knockdown efficacy via Western blotting analysis (Fig. 3A). The cells that were transfected with si-1 were designated the experimental group (si-HMGB1), whereas those transfected with a negative control siRNA served as the control group (si-NC). The effect of HMGB1 on cell proliferation was assessed via the CCK8 assay, and the results revealed a significant reduction in the absorbance of CRC cells at 450 nm following HMGB1 downregulation (Fig. 3B), indicating the inhibition of CRC cell proliferation. Consistent results were obtained from the colony formation assay, where HMGB1 downregulation led to a significant reduction in both the size and number of CRC cell colonies formed (Fig. 3C).

To evaluate the impact of HMGB1 on cell migration and invasion, we performed Transwell assays. Transwell plates with or without Matrigel were used to measure cell invasion and migration abilities, respectively. The migration assay revealed a significant decrease in the number of migrating cells in the si-HMGB1 group (Fig. 3D). To assess invasion, Transwell chambers that were coated with Matrigel were utilized, and the results revealed a notable reduction in the number of invading cells in the si-HMGB1 group (Fig. 3D). Additionally, we investigated the cell adhesion capacity in an adhesion assay. The results revealed a significant increase in the number of cells that adhered to the 96-well plates in the si-HMGB1 group (Fig. 3D). These findings suggest that the downregulation of HMGB1 decreases cell migration and invasion while increasing cell adhesion.

### HMGB1 contributed to promoting metastasis in CRC cells by regulating the EMT process

**Fig. 4:**
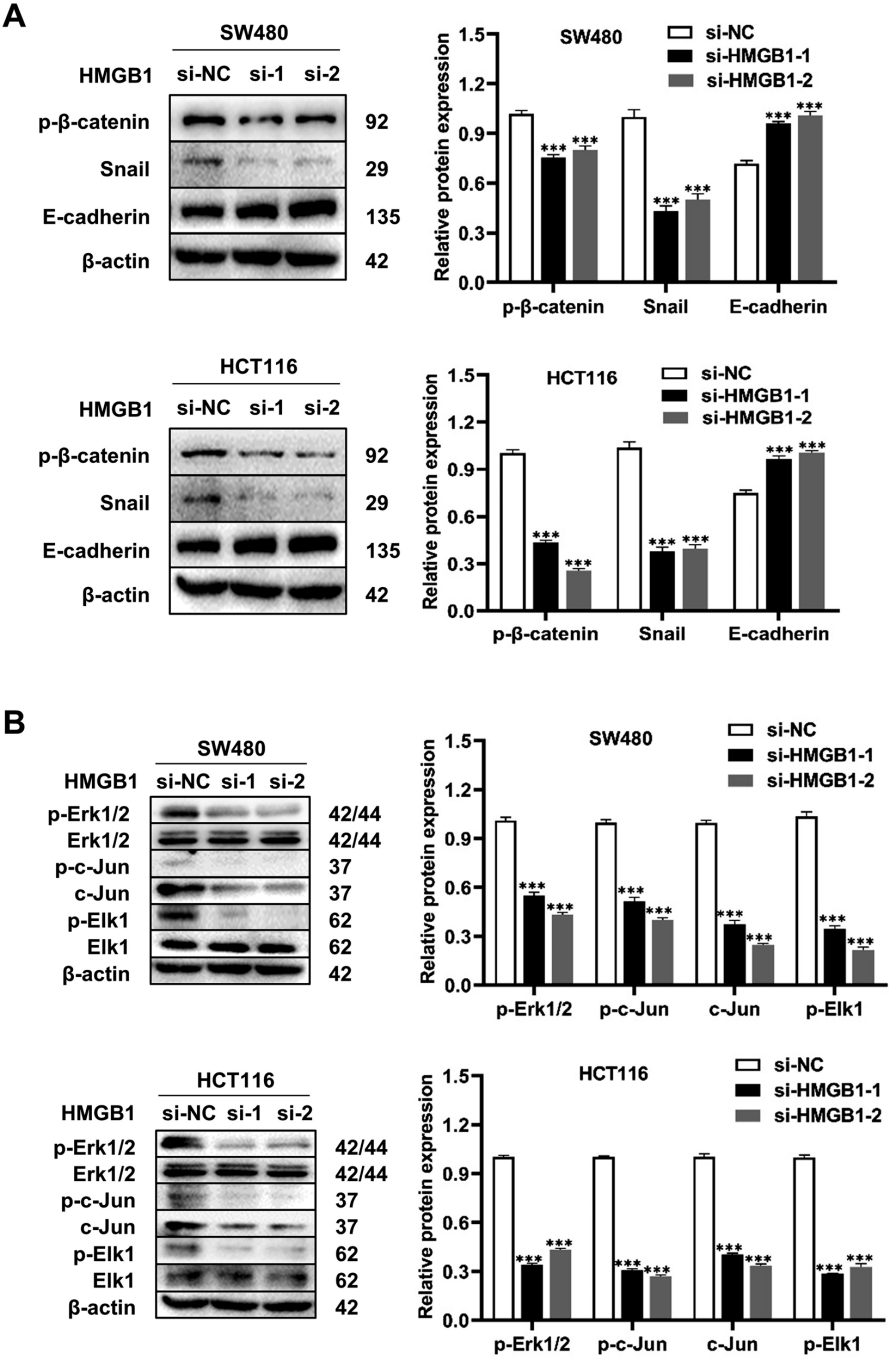
The mechanism of HMGB1 in promoting CRC. **A** Expression levels of p-β-catenin, snail and E-cadherin were detected by Western blotting in SW480 cells (upper panel) and HCT116 cells (lower panel). Semiquantitative analysis revealed that in HMGB1-knockdown groups, p-β-catenin and snail expression significantly decreased, while E-cadherin expression significantly increased (***p < 0.001); **B** Levels of p-Erk1/2, p-c-Jun, c-Jun and p-Elk1 were detected by Western blotting in SW480 cells (upper panel) and HCT116 cells (lower panel). Semiquantitative analysis revealed that in HMGB1-knockdown groups, p-Erk1/2, p-c-Jun, c-Jun and p-Elk1 levels significantly decreased (***p < 0.001). All graphs are drawn from the mean ± SD of three independent replicates.

Our research revealed a correlation between HMGB1 expression and lymph node and distant metastasis in CRC cells. HMGB1 downregulation has been shown to inhibit cell migration and invasion. To explore the underlying mechanism, we further investigated the impact of HMGB1 on the EMT process. E-cadherin, p-β-catenin, and Snail are well-established markers of EMT. Using two distinct siRNA sequences, we downregulated HMGB1 expression in SW480 and HCT116 cells. Compared with the si-NC group, both the si-1 and si-2 groups exhibited decreased expression of p-β-catenin and Snail and increased expression of E-cadherin (Fig. 4A). In the xenograft tumors, we verified E-cadherin expression through IHC staining, and the results revealed that E-cadherin expression was also elevated in the KD group, confirming that this finding was also applicable in vivo (Fig. 6D). Collectively, our findings confirm that HMGB1 promotes metastasis in CRC cells by regulating EMT.

### HMGB1 may influence the malignant biological behaviour of CRC via the MAPK pathway

Research has highlighted the significant role of the MAPK signaling pathway in the initiation and progression of CRC [15, 16]. To explore the underlying mechanism, we examined key molecules within the classical MAPK pathway, including Erk1/2, Elk1, and c-Jun [17]. Our results revealed that, compared with those in the si-NC control group, the phosphorylation of these proteins was markedly suppressed in the si-1 and si-2 groups, which exhibited reduced HMGB1 expression in both the SW480 and HCT116 cell lines (Fig. 4B). These findings were further validated by IHC staining of xenograft tumors (Fig. 6D). Together, these results suggest that HMGB1 may exert its effects via the MAPK pathway.

### HMGB1 promotes the progression of CRC by downregulating the expression of DDX3

**Fig. 5:**
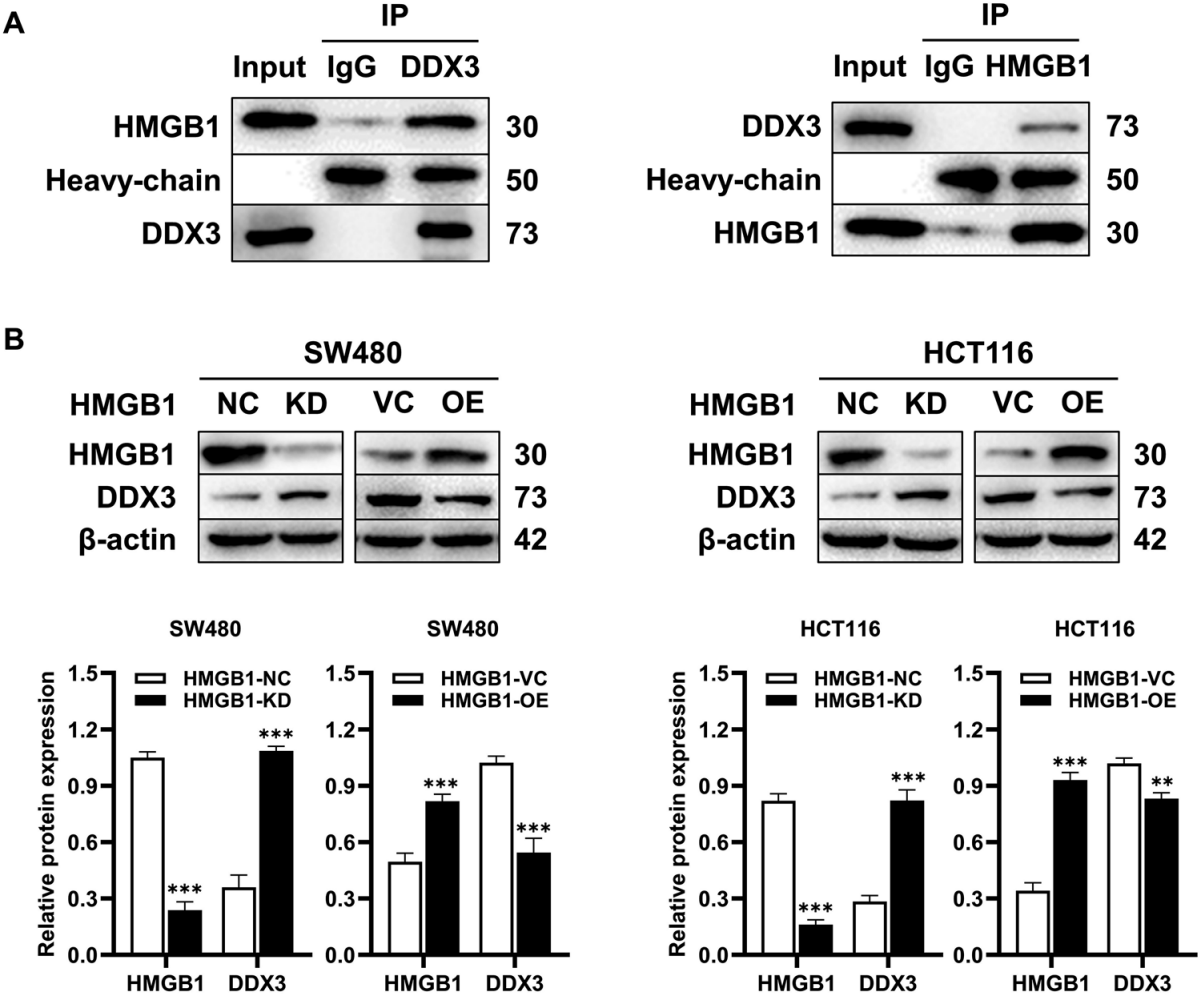
The interaction between HMGB1 and DDX3. **A** The Co-IP experiment results indicated that when anti-DDX3 was added to the total protein, the presence of HMGB1 could be detected in the immune complex, and vice versa; **B** SW480 and HCT116 cells were infected with lentivirus, respectively. The expression of DDX3 was detected subsequently, indicating a negative correlation between the expression of DDX3 and HMGB1. (***p < 0.001, **p < 0.01). All graphs are drawn from the mean ± SD of three independent replicates. Abbreviation: EV empty vector, KD knockdown, NC negative control, OE overexpression.

Our previous research revealed that DDX3 inhibits the onset of CRC via the MAPK pathway. In the present study, we demonstrated that HMGB1 also exerts its effects through the MAPK pathway, suggesting a potential interaction between HMGB1 and DDX3. To validate this interaction, we conducted Co-IP experiments, and the results revealed the possible occurrence of this interaction (Fig. 5A). Additionally, we infected SW480 and HCT116 cell lines with lentivirus to generate HMGB1-overexpressing and HMGB1-knockdown cell lines. The infection efficiency was assessed by Western blotting. We subsequently examined DDX3 expression in these cells by Western blotting analysis, which revealed a negative correlation between DDX3 expression and HMGB1 expression, further supporting the potential interaction between the two proteins (Fig. 5B).

### The downregulation of HMGB1 expression inhibited cell proliferation in vivo

**Fig. 6:**
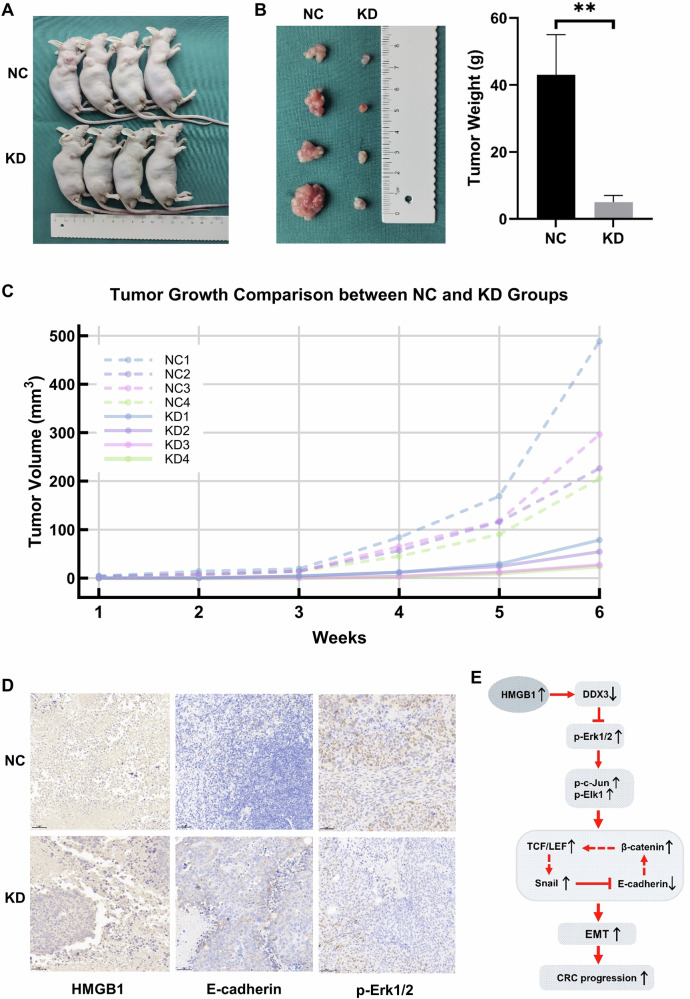
HMGB1 knockdown suppresses in vivo growth of CRC xenografts (SW480 model). **A** Images of nude mice with subcutaneous tumors; **B** Images of the excised subcutaneous transplanted tumor (left panel) and a bar graph comparing the tumor tissue weight (right panel), after 6 weeks, the tumor showed lower weight in KD group (**p < 0.01); **C** A line graph showing the changes in subcutaneous tumor volume over the growth weeks in 4 NC group nude mice and 4 KD group nude mice; **D** Representative images of IHC staining of HMGB1, E-cadherin and p-Erk1/2; **E** Schematic model illustrating how HMGB1 downregulates DDX3 to promote CRC progression by regulating E-cadherin and β-catenin signaling through the MAPK pathway. Abbreviation: CRC colorectal cancer, EMT epithelial-mesenchymal transition, KD knockdown, NC negative control.

To investigate the impact of HMGB1 on CRC cell function in vivo, we conducted a nude mouse tumorigenesis assay to determine whether the decreased expression of HMGB1 inhibited tumor growth in vivo. SW480 cells with HMGB1 knockdown, along with negative control cells, were subcutaneously injected into nude mice (Fig. 6A). Compared with the NC group, the KD group exhibited smaller xenograft tumor volumes and lighter tumor weights after 6 weeks (Fig. 6B, C). The xenograft tumor tissues were subsequently embedded in paraffin and subjected to IHC staining. The results revealed that HMGB1 knockdown in vivo upregulated E-cadherin expression and downregulated p-Erk1/2 levels, which was consistent with the Western blotting results obtained in vitro (Figs. 6D and 4). To ensure reproducibility, we conducted additional in vivo experiments using an HMGB1-knockdown HCT116 model (Supplementary Fig. 1A, B), the results of which were consistent with the SW480 data (tumor growth inhibition *p* < 0.01). It should be noted that tail vein injection of SW480 cells did not yield detectable pulmonary metastases after six weeks, likely due to the low metastatic potential of this cell line under the present experimental conditions.

## Discussion

High mobility group (HMG) proteins play critical roles in various intracellular and extracellular functions, significantly contributing to biological processes. These proteins are categorized into three superfamilies: HMG AT Hook (HMGA), HMG nucleosome binding (HMGN), and HMGB proteins [7]. Among these proteins, HMGB1 belongs to the HMGB family and functions as a DNA partner that regulates nuclear homeostasis and genomic stability [9]. HMGB1 directly interacts with damaged DNA and is involved in several DNA repair pathways, including base excision repair, mismatch repair, nucleotide excision repair, and double-strand break repair. Recent studies have linked HMGB1 to the promotion of malignant behaviors in various cancers, including liver cancer, ovarian cancer, and non-small cell lung cancer, through the modulation of mitochondrial function, EMT, autophagy, and other pathways [11, 13, 18]. In their clinical research, Wenjia Zhang et al. reported that high HMGB1 expression may be associated with the development of CRC, although the exact underlying mechanism remains unclear [19]. Motivated by these findings, we aimed to explore the relationship between HMGB1 and CRC. Initially, bioinformatics analysis indicated that HMGB1 expression was higher in CRC patients than in control individuals. We subsequently investigated the correlations among HMGB1 expression, clinicopathological parameters, and patient prognosis using TMA analysis. Finally, cell-based experiments confirmed that HMGB1 might promote CRC cell proliferation, invasion, and adhesion, potentially through the regulation of the EMT and MAPK pathways.

The Ras/Raf pathway is widely known to respond to extracellular signals, such as growth factors, subsequently activating the MAPK cascade and transmitting extracellular signals to the nucleus [20]. The small G proteins encoded by the Ras genes (H-Ras, K-Ras, and N-Ras) bind to the cell membrane and exhibit GTPase activity. Upon activation, Ras mediates the phosphorylation and activation of Raf proteins (A-Raf, B-Raf, and C-Raf/Raf1) [21]. These Raf proteins then sequentially relay signals to MEK (MEK1 and MEK2) and Erk (Erk1 and Erk2) proteins. Erk translocates into the nucleus, where it modulates transcription factors such as Elk1, c-Jun, and NF-κB, promoting the transcription of genes that are involved in cell cycle regulation and proliferation [17, 22]. Within the multistep genetic model of CRC, K-Ras gene mutations have emerged as a pivotal event. Research by Amato et al. demonstrated that HMGB1 can regulate tumor onset and maintenance by binding to G4 DNA structures, influencing telomerase activity and K-RAS gene transcription [23]. Therefore, we hypothesized that HMGB1 may contribute to the development of CRC. Our experiments further support this hypothesis by showing that HMGB1 modulates the malignant biological behaviors of CRC through the MAPK pathway.

When immune or tumor cells are stimulated by both internal and external factors, HMGB1 can be secreted into the extracellular space, where it binds to various receptors on the cell membrane, such as the advanced glycation end product (RAGE) receptor, Toll-like receptors, CXCL4, TIM-3, and others. This interaction activates downstream signaling pathways, including the NF-κB, PI3K and IRF3 pathways [24–26]. Zhu et al. demonstrated that in CRC, the interaction between HMGB1 and RAGE promotes the expression of signaling molecules that are related to EMT. In our study, we observed that HMGB1 knockdown inhibited the expression of β-catenin and Snail while increasing the expression of E-cadherin, suggesting that HMGB1 may play a role in the EMT process [27].

Several HMGB1-targeting inhibitors, including HMGB1-neutralizing antibodies, A box proteins, soluble RAGE (sRAGE), platinum-based agents, ethyl pyruvate, glycyrrhizin, and quercetin, have been explored in experimental studies investigating antitumour therapies [28–31]. Research has demonstrated that sRAGE effectively blocks the HMGB1-RAGE signaling pathway by inhibiting the RAGE receptor [32]. Platinum agents, such as oxaliplatin and cisplatin, promote the aggregation of HMGB1 in the nucleus by stabilizing its binding to the double helix structure [33]. Our study suggests that downregulation of HMGB1 can inhibit the proliferation and metastasis of CRC cells.

In our previous research, we demonstrated that DDX3 inhibits the progression of CRC via the MAPK pathway [14]. Since the HMGB1 protein also plays a role in this pathway, we further investigated the relationship between HMGB1 and DDX3 (Fig. 6E). Co-IP experiments confirmed a direct interaction between HMGB1 and DDX3 (Fig. 5A). Additionally, we observed reciprocal regulation between the expression of HMGB1 and that of DDX3 across different cell lines (Fig. 5B). Specifically, when HMGB1 expression was knocked down, the level of DDX3 expression increased, suggesting that HMGB1 may suppress DDX3 expression. While the precise molecular cascade (e.g., whether HMGB1 promotes DDX3 degradation via ubiquitination) requires further investigation, our current data support a model in which HMGB1-driven DDX3 suppression releases MAPK pathway inhibition, thereby driving tumor progression. The discovery of this HMGB1-DDX3-MAPK regulatory axis provides novel insights into CRC pathogenesis.

While our experimental data revealed a functional interaction between the DDX3 and HMGB1 proteins in CRC progression, bioinformatics analyses of public datasets revealed no significant correlation between their mRNA expression levels. This apparent discrepancy may be explained by several factors: (1) posttranslational regulation being the primary mode of interaction between DDX3 and HMGB1 (as evidenced by co-IP), (2) tumor heterogeneity diluting cohort-level transcriptional correlations, and (3) limitations in current databases where matched DDX3/HMGB1 protein expression data remain scarce. We hypothesize that changes in the subcellular localization of HMGB1, which is a nuclear protein, across different cellular compartments (e.g., between the nucleus and cytoplasm) might affect its interaction with DDX3 and the stability of DDX3, thereby influencing its translational regulation. While we have made some initial findings in this regard, further experimental validation is needed to confirm these observations.

## Conclusions

This study demonstrated that HMGB1 expression is upregulated in CRC, with elevated levels correlated with poor prognosis in CRC patients. Knockdown of HMGB1 inhibited cell proliferation and metastasis, potentially through the modulation of DDX3 expression and the involvement in the MAPK pathway and the EMT process. These findings suggest that targeting HMGB1 may offer novel therapeutic strategies for CRC patients.

### Limitations and future directions

While our study provides insights into the role of HMGB1 in CRC progression, the exact mechanisms by which the MAPK pathway and EMT are regulated remain unclear, and the interaction between HMGB1 and DDX3 requires further exploration. Future work should include in vivo models, larger clinical studies to validate the prognostic value of HMGB1, and investigations into HMGB1-targeting inhibitors for therapeutic potential. Additionally, understanding the subcellular localization and regulatory interactions of HMGB1 could reveal novel mechanisms and inform combination therapies for the treatment of patients with CRC.

## Supplementary information

Supplementary Figure 1 HMGB1 knockdown suppresses in vivo growth of CRC xenografts (HCT116 model)

Supplementary Table 1 Detailed information on HMGB1 knockdown and overexpression lentiviral vectors

## Data Availability

The data are available from the corresponding author upon reasonable request.
