# Supplementary material for: HMGB1 downregulates DDX3 to activate the MAPK pathway, promoting the progression of colorectal cancer
Source: Cancer Gene Ther. 2025 Sep 20;32(12):1307–18. doi: 10.1038/s41417-025-00963-z (PMC12702777; doi:10.1038/s41417-025-00963-z)
Supplement: Supplementary file 1 — Supplementary Figure 1 HMGB1 knockdown suppresses in vivo growth of CRC xenografts (HCT116 model) [file 41417_2025_963_MOESM1_ESM.docx]

**
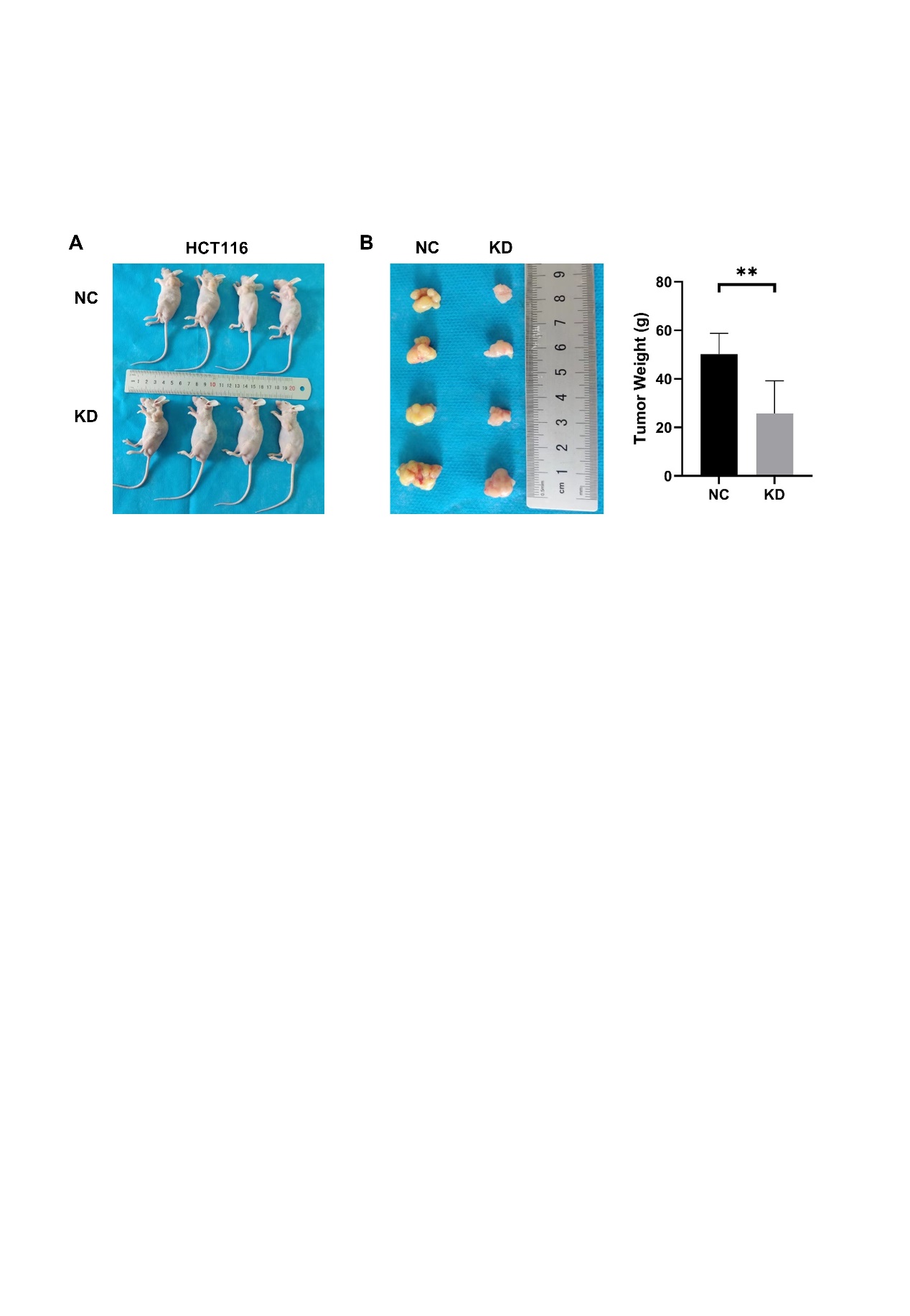
**

**Supplementary Figure 1 HMGB1 knockdown suppresses in vivo growth of CRC xenografts (HCT116 model)**

A: Images of nude mice with subcutaneous tumors; B: Images of the excised subcutaneous transplanted tumor (left panel) and a bar graph comparing the tumor tissue weight (right panel), after 6 weeks, the tumor showed lower weight in KD group (**p<0.01). Abbreviation: CRC, colorectal cancer; KD, knockdown; NC, negative control.
