# Supplementary material for: HMGB1 downregulates DDX3 to activate the MAPK pathway, promoting the progression of colorectal cancer
Source: Cancer Gene Ther. 2025 Sep 20;32(12):1307–18. doi: 10.1038/s41417-025-00963-z (PMC12702777; doi:10.1038/s41417-025-00963-z)
Supplement: Supplementary file 2 — Supplementary Table 1 Detailed information on HMGB1 knockdown and overexpression lentiviral vectors [file 41417_2025_963_MOESM2_ESM.docx]

**Supplementary Table 1 Detailed information on HMGB1 knockdown and overexpression lentiviral vectors**

| Parameter | HMGB1 knockdown | HMGB1 overexpression |
| --- | --- | --- |
| shRNA Sequence | Sense: 5'-CCCGUUAUGAAAGAG  AAAUUUCAAGAGAAUUUCUCU  UUCAUAACGGGTTTTTG-3'  Antisense: 5'-UUCUCCGAACGUGUC  ACGUUUCAAGAGAACGUGACA  CGUUCGGAGAATTTTTG-3' | / |
| vector | 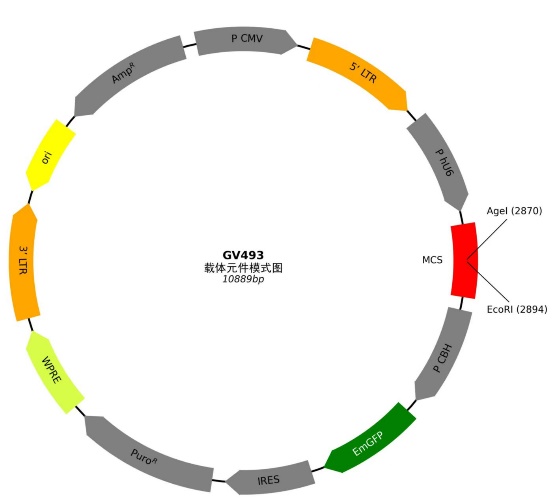 | 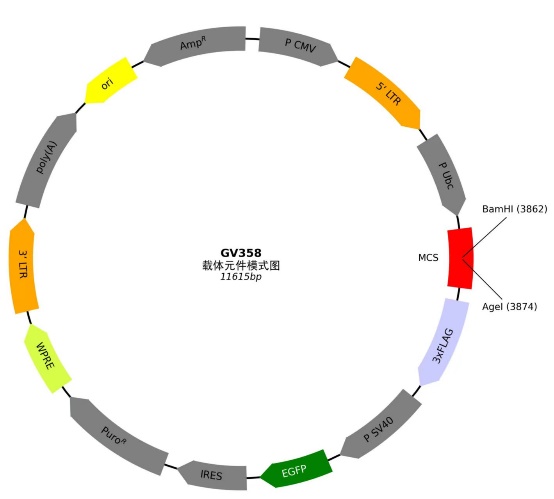 |
| Contract Number | GISL0350526 | GOSL0106841 |
| Manufacturer | GeneChem Co., Ltd. (Shanghai, China) | GeneChem Co., Ltd. (Shanghai, China) |
